# Mahi tahi (working together) and the lessons learned from implementating a concussion management pathway for community rugby in New Zealand: Haerenga (our journey)

**DOI:** 10.1016/j.jsampl.2026.100156

**Published:** 2026-09-24

**Authors:** Danielle M. Salmon, Marelise Badenhorst, Zachary Yukio Kerr, Chris Whatman, James Brown, Janelle Romanchuk, Johna Register-Mihalik, Simon Walters, Gisela Sole, Ian Murphy, Karen Rasmussen

**Affiliations:** a New Zealand Rugby, PO Box 2172, Wellington, 6140, New Zealand; b International Rugby Players Association, Dublin, Ireland; c Sport Injury Prevention Research Centre, Faculty of Kinesiology, University of Calgary, Alberta, Canada; d School of Physical Education, Sport and Exercises Sciences, University of Otago, Dunedin, New Zealand; e Sports Research Institute New Zealand, School of Sport and Recreation, Auckland University of Technology, New Zealand; f Carnegie Applied Rugby Research (CARR) Centre, Carnegie School of Sport, Leeds Beckett University, Leeds, UK; g The Division of Sport and Exercise Medicine (DiSEM), Department of Exercise, Sport and Lifestyle Medicine, Faculty of Medicine and Health Sciences, Stellenbosch University, South Africa; h Division of Physiological Sciences, Department of Human Biology, Faculty of Health Sciences, the University of Cape Town and the Sports Science Institute of South Africa, Cape Town, South Africa; i Matthew Gfeller Sport-Related Traumatic Brain Injury Research Center, Department of Exercise and Sport Science, University of North Carolina at Chapel Hill, Chapel Hill, USA; j Department of Exercise and Sport Science, University of North Carolina at Chapel Hill, Chapel Hill, USA; k Centre of Health, Activity, Rehabilitation Research, School of Physiotherapy, University of Otago, Box 56, Dunedin, 9054, New Zealand

**Keywords:** Implementation science, Systems thinking, Knowledge translation, Intervention adaptation

## Abstract

Concussion guidelines are widely available, but implementing them consistently in community sport remains difficult. Common barriers include limited access to medical care, reliance on volunteers, fragmented communication, and sporting cultures that may discourage reporting. In response, New Zealand Rugby developed and refined over multiple seasons, a community Concussion Management Pathway (CMP), to operationalise World Rugby's principles of ‘Recognise and Remove’ in real-world community settings.

This editorial reflects on lessons learned from the design, implementation, and iterative refinement of the CMP over multiple seasons. Rather than evaluating effectiveness, we focus on the practical realities of implementation within a complex community sport system. Key lessons learned include the importance of co-design with stakeholders, adapting elite-derived processes to community capacity, balancing standardisation with flexibility, and recognising the complexity associated with baseline testing, technology use, and navigating primary healthcare pathways. The role of education, role clarity, and systems-level communication in shaping concussion reporting and management behaviours are also explored.

Our experiences highlight that in community sport concussion interventions must be treated as living systems, requiring ongoing adaptation to local context, resources, and cultures. The lessons outlined provide transferable insights for researchers, sporting organisations, and policy-makers seeking to design, implement, or scale concussion prevention and management initiatives across community sport.

## 1 Introduction

Concussions are a global concern for players, coaches [1], medical professionals, and administration staff across sport, with many governing bodies responding by developing guidelines and recommendations involving education initiatives, policy reform, rule changes, and evidence-informed interventions [2,3]. In 2022, World Rugby responded by updating their Return to Play (RTP) guidelines for both the elite and community games, where the community game currently has a 21-day stand-down period (SDP) [4]. However, the existence of these guidelines alone does not mean that people will adhere to them [1,5]. Uptake of concussion guidelines into routine practice in community sport remains challenging due to under-reporting, difficulties accessing diagnosis and medical clearance assessments in primary care, limited mechanisms for tracking recovery, fragmented stakeholder communication, and variability in resourcing and role clarity. [[1], [2], [3],^6^,^7^]. Implementation is further complicated by the gap between formal recommendations and real-world practice, where performance priorities intersect with medical, sociocultural, and logistical factors [[8], [9], [10], [11]].

Unlike professional environments that have access to resources such as video review and pitch-side match day doctors [12], community rugby relies heavily on volunteer staff and often lacks immediate access to medical professionals, making timely and accurate pitch-side recognition and diagnosis of concussions more difficult [13,14]. These challenges are not unique to community rugby and have been reported in other community sports in Ireland [15], British American football [16] and Gaelic football [17]. Cultural factors, such as pressure to RTP(16), player stoicism, and limited understanding regarding the potential for long-term sequelae of concussions, can undermine compliance with best practice guidelines [7,9]. Furthermore, resources, training, and adherence to consistent SDP protocols varies across the diverse landscape of clubs, cultures and age groups [5,6,17,18]. Together, these factors highlight the need for accessible, evidence-based education and management systems, tailored to the community contexts in which they are used, to support the use of guidelines in everyday practice [9,18,19].

In 2012, World Rugby introduced the Head Injury Assessment (HIA) process for the elite game to standardise concussion identification and medical management [12]. The HIA protocol utilises the Specialised Concussion Rugby Management (SCRM) App to support pitch-side, post-match, and 48-h medical assessments by a doctor for the diagnosis of a concussion [12,20]. This protocol requires that players complete a pre-season baseline assessment, which gives clinicians an individual reference point when interpreting a post-injury assessment; it is not used as a stand-alone test to diagnose concussion [20]. While the implementation of the HIA protocol has successfully improved pitch-side recognition and concussion management [12], it is only applied to the elite level of the game. In 2017, NZR sought to determine whether a similar process could be implemented in the community game [21]. To achieve this, NZR explored how the key decision points (baseline assessment, suspected concussion recognition and 48 h diagnosis and clearance assessment) could be supported by an App similar to SCRM, while acknowledging the realities of community rugby. This work led to the development of NZR's Concussion Management Pathway (CMP), designed to operationalise World Rugby's community concussion guidelines [21]. Over the course of implementation (2017–2022), several components required substantial modification (baseline assessment, information flow for the logging of a suspected concussion and the protocol for concussion diagnosis and medical clearance), highlighting the limitations of directly translating elite-level systems into community sport.

This opinion piece describes how the CMP was implemented and refined across the New Zealand community rugby system [21]. Rather than discussing effectiveness, this editorial examines the realities of implementing concussion guidelines within a complex community sport system. The lessons learned through the implementation of NZR's CMP offer valuable insights for others seeking to develop, implement, or scale concussion management initiatives in community sport and beyond. Fig. 1 shows the season-by-season cycle used to gather feedback, revise the pathway, and test changes. The four lessons that follow are organised around this cycle and are intended to help others plan, implement, and sustain community concussion programmes.

**Fig. 1:**
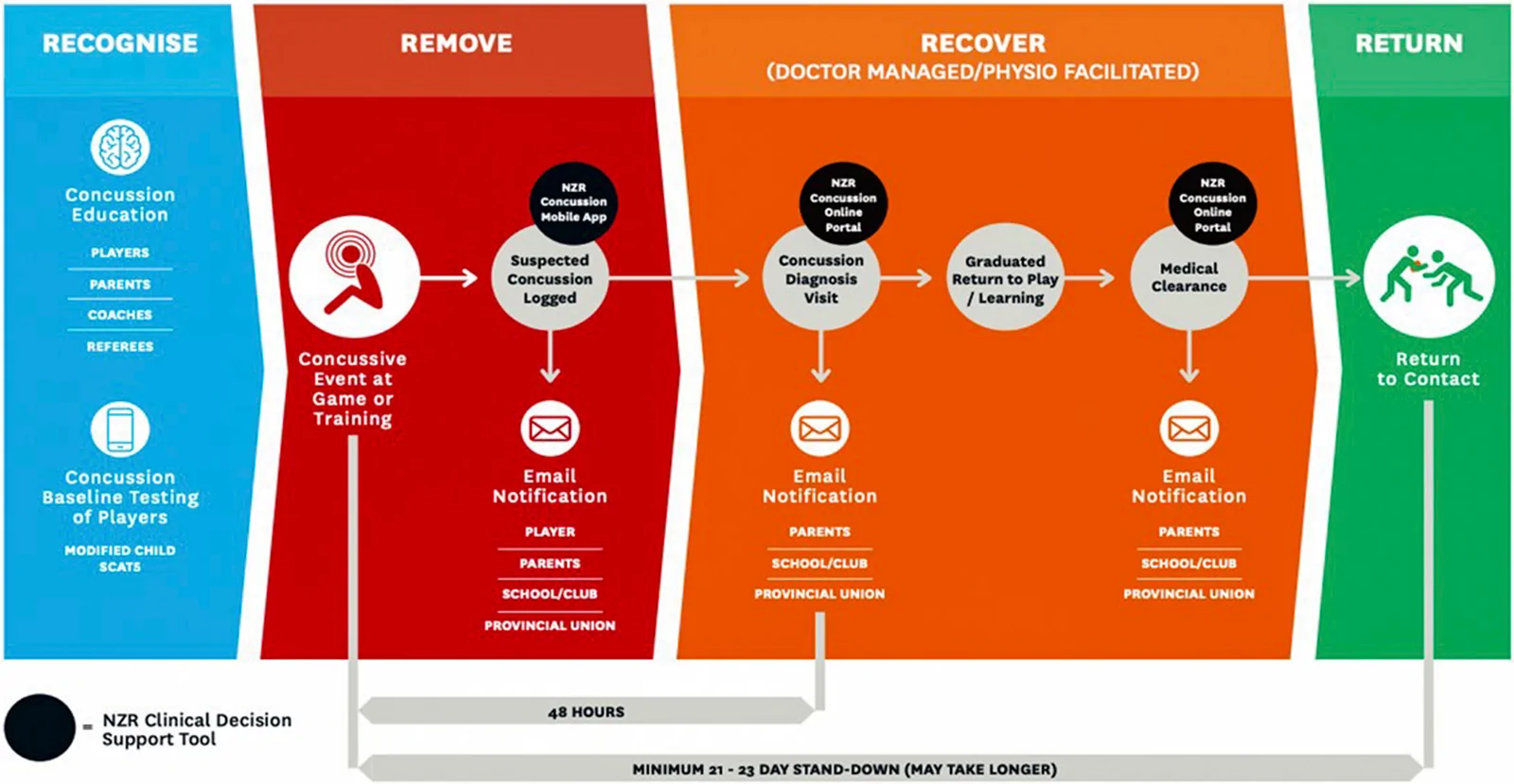
A detailed outline of the New Zealand Rugby’s Concussion Management Pathway from baseline testing to return to contact.

## 2 Designing a concussion management pathway for community rugby

A systems thinking approach was employed to develop the CMP and guide evaluation and refinement, ensuring the process was responsive to community needs while promoting positive changes in concussion attitudes and reporting behaviours [22,23]. This approach considers that decisions and actions across occur all levels of the sporting system, from governing bodies to individual players [23,24]. Adopting and sustaining implementation of interventions successfully in the real-world settings depends on collaboration with the community [9,18,19]. Rather than viewing an intervention as a fixed program, it should be continuously refined through iterative and ongoing engagement with local stakeholders, including players, coaches and union representatives, to ensure that the program remains relevant and appropriate for the local context [19]. Regular consultation allows key program components to be adapted to the unique cultural, resource, and logistical realities of the setting in which it is implemented.

Rather than replicate the elite HIA process, the CMP focused on three points at which management commonly fails in community rugby: immediate removal from play, clinical follow-up within 36–48 h, and medical clearance before returning to contact training and playing. [14,21]. Acknowledging the differences between the elite and community games, the NZR App was designed to be flexible and practical for a range of users and settings. It enabled teams to access functions such as baseline testing, logging a suspected concussion, and GRTP management, while also allowing teams with limited medical or administrative support to use the core functions to log a suspected concussion [21,25].

## 3 NZR's concussion management pathway

A detailed diagram outlining the steps in the pathway is provided in Fig. 2. The first phase of the CMP included comprehensive concussion education for players, parents, coaches, and referees, alongside pre-season baseline testing for players [6,26]. Baseline testing involves assessing a players cognitive function and reaction skills. Following a suspected concussion, post-injury results can be compared with the player's baseline scores to support the general practitioner's clinical diagnosis decisions regarding return-to-play. The subsequent steps of the CMP are as follows:

1. When a suspected concussion occurred during a match or training, that incident was logged using the NZR App, typically by the team manager or physiotherapist.
2. The NZR App then generated an email notification to the player, their parents/caregivers, coach, school or club, and provincial union (PU). The email to the player and their parents/caregivers contained a unique code that they provided to the General Practitioners (GPs, the equivalent to physicians in other countries). This code gave the GP access to the player's baseline concussion assessment in the secure GP Portal. The GPs could review baseline scores, repeat the assessment and obtain guidance for diagnosis and medical clearance.
3. Players diagnosed with a concussion were required to observe a SDP, incorporating graduated RTP (GRTP) protocol.
4. Players were asked to obtain medical clearance from a GP after completing the non-contact stage of the GRTP, prior to resuming contact training. An email notification was sent to relevant stakeholder to confirm that the player had been cleared to return [21,25].

**Fig. 2:**
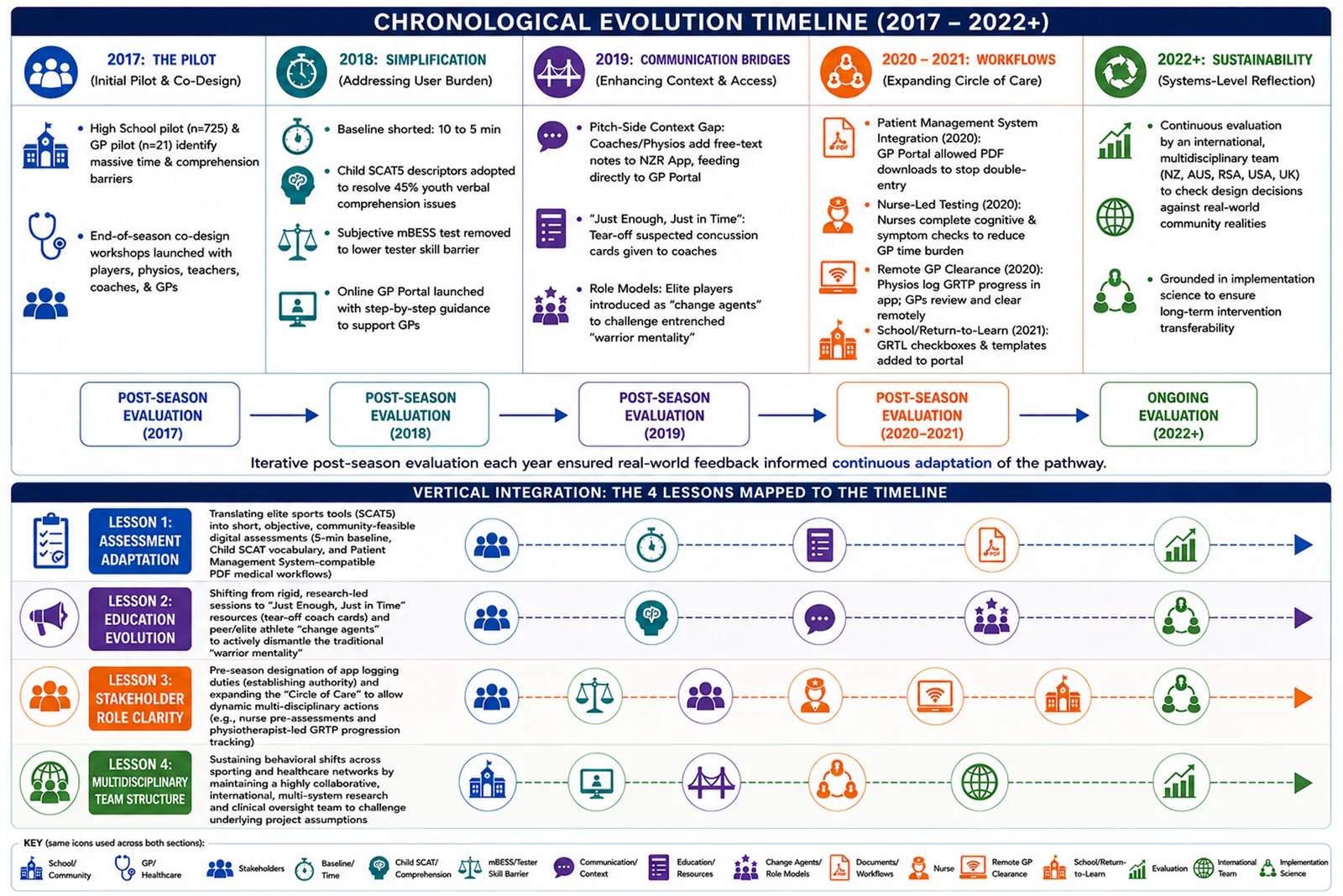
A detailed outline of the evolution of New Zealand Rugby's Concussion Management Pathway over the study time period 2017 -2022 and the four key lessons learned over this time period.

## 4 Lessons learned & adaptations made to the concussion management pathway

NZR co-designed the pathway and resources alongside community stakeholders (NZR App, the GP Portal, and pre-season concussion education). This co-design followed a cyclical, season-by-season pattern: user feedback was collected at the end of each season, the pathway and supporting hardware (NZR App and GP Portal) were refined, then re-evaluated the following season [11]. Fig. 2 summarises this cycle and the adaptations made between 2017 and 2022, alongside the four main lessons learned:

1. Translating the SCAT5 baseline assessment for high schools and primary care
2. Evolution of Concussion Education & Resources
3. The Concussion Management Pathway Provided Role Clarity
4. Building the Right Team to Support Implementation

## 5 Lesson 1: Translating the SCAT5 for baseline assessments in high schools and primary care

In 2017, pre-season baseline testing using the SCAT5, was piloted with 725 high-school players, and 21 GPs used the GP Portal to support post-injury assessment [13,27]. The SCAT5 had been developed for trained clinicians in better-resourced settings and proved difficult to use in schools and primary care. End-of-season workshops with players, teachers, coaches, physiotherapists, GPs, sports medicine doctors, and NZR staff identified the changes needed for the 2018 season [13,27]. Table 1 and Table 2 summarise the practical problems, adaptations, and lessons.

**Table 1:**
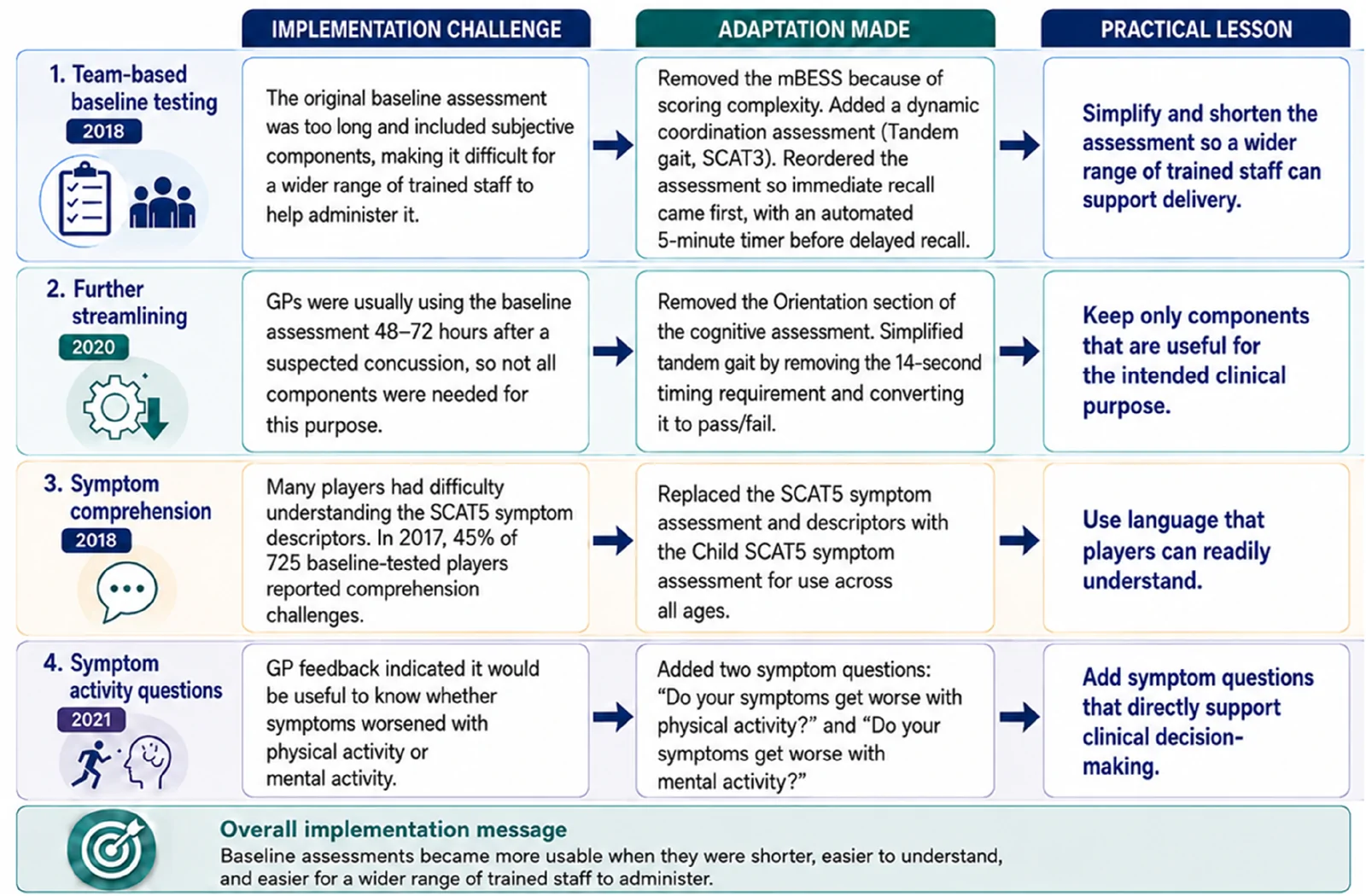
Adapting baseline SCAT Assessment for the NZR Commonuty Concussion Management Pathway. Key implementation challenges, adaptations and lessons learned from New Zealand community rugby.

Note: GP = general practitioner; mBESS = Modified Balance Error Scoring System; NZR = New Zealand Rugby; SCAT3 = Sport Concussions Assessment Tool 3rd Edition; SCAT5 = Sport Concussions Assessment Tool 5th Edition.

**Table 2:**
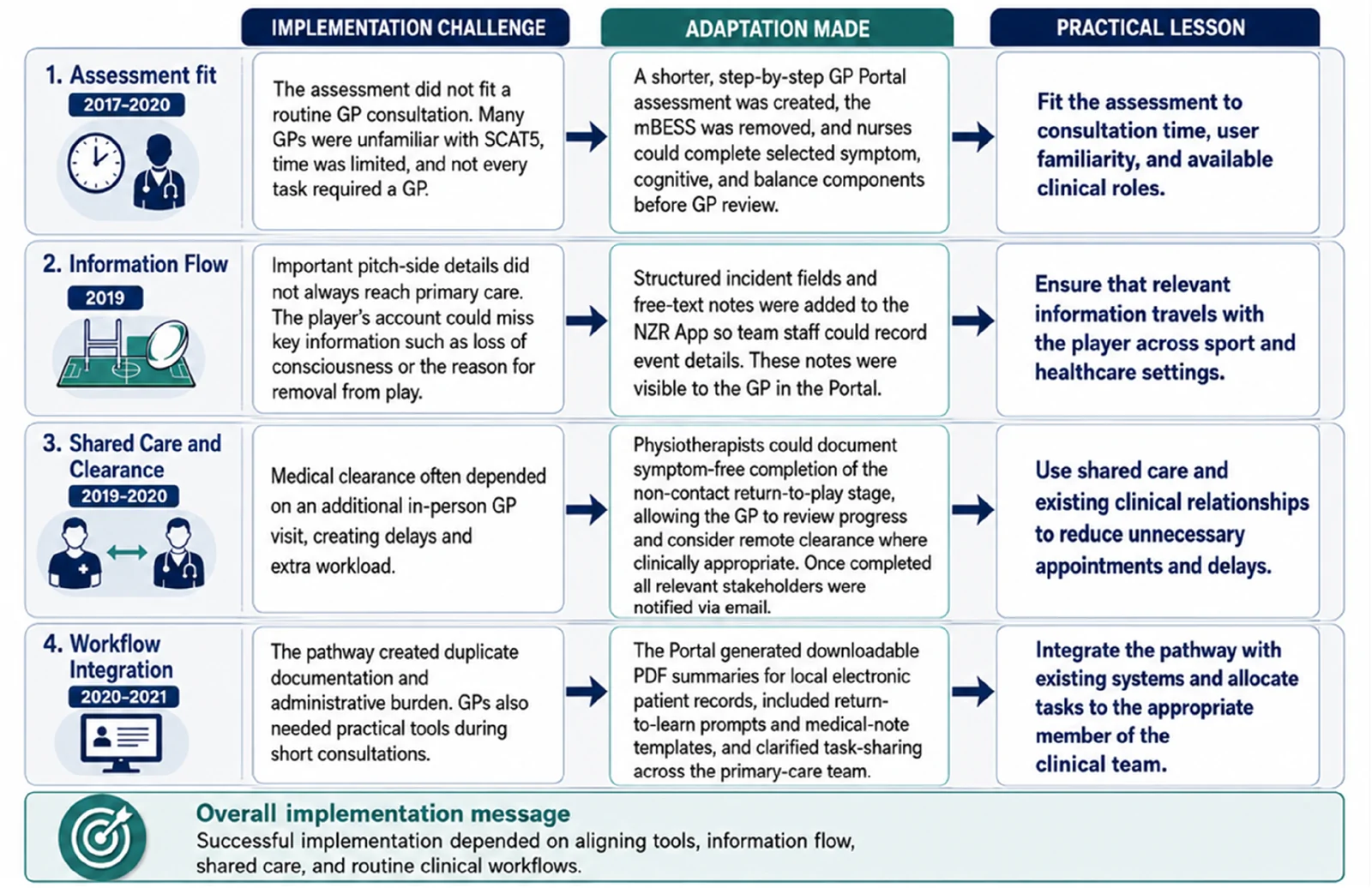
Implementing the NZR Community Concussion Management Pathway in primary care: Key implementation challenges, adaptions and lessons from New Zealand community rugby.

Abbreviations: CMP= Concussion management pathway; GP= General practitioner; GRTP = Graduated return to play; mBESS= Modified Balance Error Scoring System; NZR= New Zealand Rugby; PDF = portable document format.

### 5.1 Adapting the baseline assessment

Key feedback from stakeholders during the 2017 post-season interviews highlighted the need to shorten the baseline assessment, improve player understanding of the testing process, and simplify administration to enable a wider range of non-medical stakeholders to assist with baseline testing (ie. removal of subjective scoring of the Modified Balance Error Scoring System (mBESS)). The challenges and the changes made to the baseline testing process are outlined in Table 1. For further detail around the development of the NZR Concussion Assessment Tool (NZRCAT) please see Salmon et al., 2022 [27].

### 5.2 Adapting the SCAT5 for primary care

Throughout the project, post-season interviews and reviews provided opportunities to reflect on how the system was working in practice and where refinements were needed. Early discussions with GPs highlighted the challenges of completing components of the SCAT within routine consultation timeframes and fitting the concussion process within existing primary-care workflows. Interviews also revealed the difficulties of relying on player self-report when GPs assessed players 24–48 h after a suspected concussion. This highlighted the importance of ensuring that accurate pitch-side information could be communicated to the treating GP. As the project progressed, conversations increasingly focused on the need to “expand the circle of care; ” GPs and physiotherapists discussed how a multidisciplinary healthcare team could better support concussion management and players’ recovery. Overall, these experiences helped to identify implementation barriers and potential refinements/adjustments to improve communication, reduce clinician burden, and better support concussion care in community rugby. The lessons learned during workshops and annual reviews around navigating primary care setting can be grouped into four implementation challenges: SCAT5 unfamiliarity and time burden, information flow from pitch-side to GP consultation, limited GP access for medical clearance, poor fit with primary-care workflows. Table 2 summarises how the pathway was adapted in response. Together, these changes show that a community concussion pathway should fit routine clinical work rather than create a parallel process.

## 6 Lesson 2: Evolution of Concussion Education & Resources

The CMP is reliant on players to self-report their concussion or others recognising a player displaying symptoms. Early evaluations suggested a prevailing “warrior mentality” and notions of “brotherhood,” where athletes prioritise team success over personal wellbeing—attitudes deeply rooted in rugby culture [7,28,29]. Similarly, when coaches focused solely on winning and adhered to “tough it out” philosophies, implementing the CMP effectively was difficult [6]. A key part of the CMP was pre-season education to address various stakeholders’ concussion knowledge and attitudes [22].

Through our engagement with stakeholders, we discovered that consistently providing information was crucial. Delivering the right information at the right time—grouped according to relevance for the player/stakeholders at each stage of their journey—proved more beneficial [26]. We developed a community concussion guideline document early 2018, outlining everything stakeholders and players needed to know about concussions in rugby. Stakeholders provided feedback that they wanted to know “just enough” at “just the right time”. For example, they did not need information about the GRTP while focusing on identifying a suspected concussion. Following a review of our resources in 2019, we designed suspected concussion cards that were provided to coaches during their annual coach education RugbySmart session in tear-off bundles (Appendix 1). These cards provided coaches and team managers with critical information to support the player for the first 48hrs until they saw a GP. We linked the provision of the GRTL and GRTP information (Concussion recovery fact sheet, Appendix 2) in the NZR App to the email sent to players and caregivers following the concussion diagnosis visit. A copy of these can we found in the Appendices.

### 6.1 Education for Players

During the 2017 and 2018 seasons, the educational sessions were conducted by CMP research staff. However, player feedback indicated a preference for role models (elite players) to deliver these sessions, who could share authentic experiences that players could relate to [7,26,30]. In response, the education sessions were modified in 2019 to include “change agents” such as professional players who would resonate with athletes and coaches [30]. We also used the notion of “brotherhood/sisterhood” to encourage peer recognition of symptoms and care for teammates [7]. We highlighted that the CMP was modelled on the elite player pathway, giving community players a sense of connection to professional rugby, enhancing engagement through the programme. These changes, alongside increased media conversations from elite players about their own concussion experiences [31,32], helped challenge the entrenched warrior mentality and contributed to behavioural shifts [26].

### 6.2 Education for General practitioners

GPs were also included, receiving peer-led education—often through sports medicine doctors and GPs currently involved in the study—on how to use the GP Portal. These sessions promoted peer-to-peer learning and created champions within the medical community [33].

### 6.3 Education for Coaches

As part of the pilot work for this study, we found that 71% of players reported receiving their concussion-related information from coaches [6]. This finding highlighted that it was important to engage coaches through the RugbySmart annual coach education course where concussion content was updated in response to the coach surveys [34]. During the study period, the number of registered coaches who had been asked to complete a RugbySmart ranged from 12550 in 2018, to 8877 in 2022. We also worked with RugbySmart coach educators, integrating concussion education into practical skill components of the course. For example, when coaching tackle technique, coaches were informed that the tackle was a high-risk event for concussion and were also given information on possible concussion signs. Emphasis was placed on GRTP protocols for coaches involved with high school girls' teams, who often lacked the direct medical support available to many boys’ teams. From 2020 to 2022, only 12% of girls club/high school teams had access to a team physiotherapist compared to 64% of boys club/high school teams. Therefore, it was critical to ensure these coaches possessed sufficient knowledge of the GRTP process, as they were frequently required to guide and support players through it [7].

In 2018, a gap was identified between coaches' knowledge and actual behaviour: only 56% of coaches (n = 733) recognized that players knocked unconscious should receive immediate medical care [34]. In response, following the end of the 2018 season, the App was updated so that when team leads/coaches/physios logged a suspected concussion in the App, the next screen asked them to review a list of red flag symptoms. If any of the red flag symptoms were selected, a pop-up notification immediately advised them that the player needed immediate medical attention (Fig. 3).

**Fig. 3:**
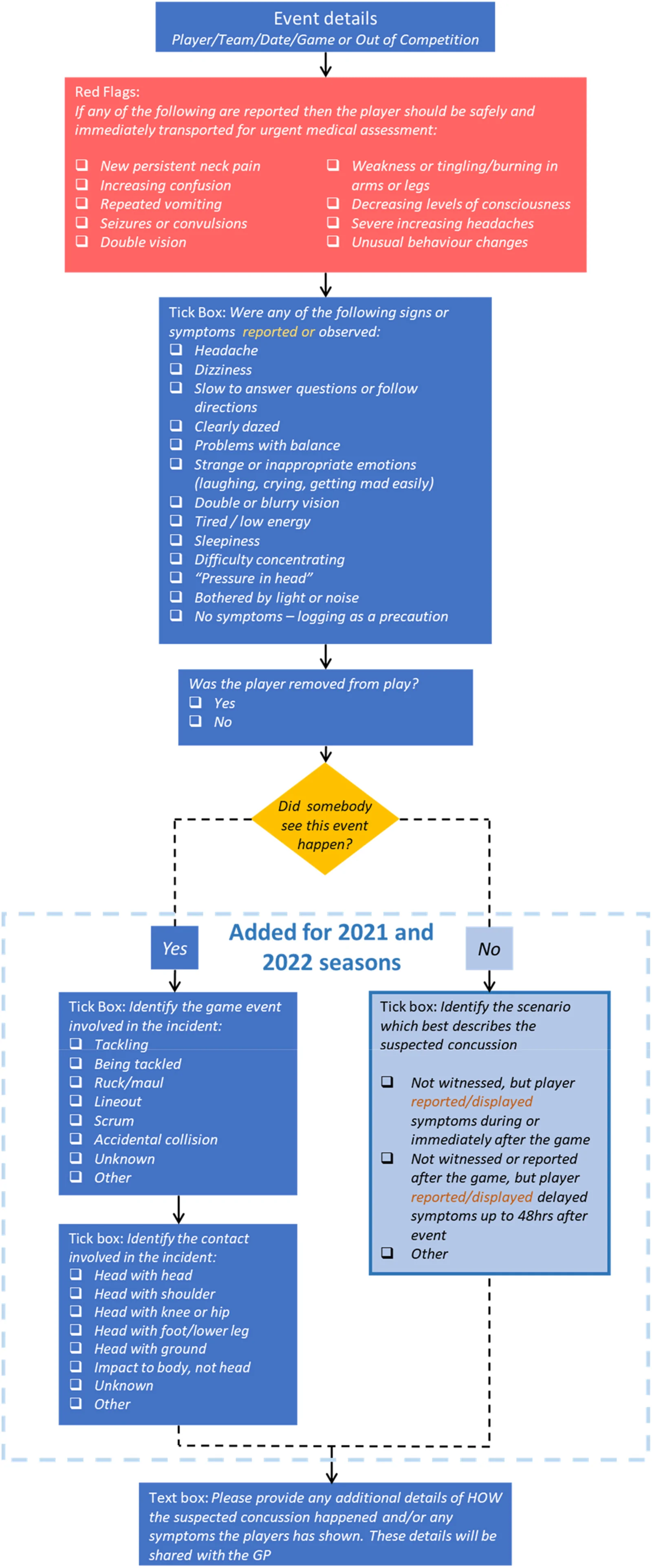
A detailed description of data flow process in the NZR App to log a suspected concussion which was completed by the team lead/medic/physiotherapist.

## 7 Lesson 3: The Concussion Management Pathway Provided Role Clarity

Clearly defined roles and responsibilities are important for effective concussion management. Yet, many stakeholders within community rugby lacked clarity about their own duties as well as those of others [11,35]. Comprehensive CMP education ensured all stakeholders were informed about consistent processes with clearly defined roles for each individual within the individual team contexts.(Salmon et al., 2023). At the start of each pre-season, every team involved in the study nominated a designated individual (physiotherapist, coach, team medic, or team manager) responsible for logging suspected concussions on the NZR App. Identifying this individual clarified their role for concussion reporting [11]. Logging the concussion also ensured all stakeholders were notified that caregivers had been informed via email of the player's removal for a suspected concussion, streamlining the communication process.

Physiotherapists/team leads had previously reported challenges when interacting with coaches, players, and parents (for example, when removing a player from the field), highlighting the need for tools to support their authority and enable them to make sound decisions [36]. In this context, the CMP, and the NZR App supported physiotherapists by providing a clear and straightforward pathway, outlining specific actions to be taken [36]. Once these steps were completed, it triggered additional support from the union and NZR. However, the system needed to be flexible enough to accommodate varying levels of team capacity, while ensuring critical requirements for safe community-level concussion management were met. For example, a team physiotherapist could coordinate baseline sessions with a senior men's team. Whereas in a high school setting, the teacher in charge of the rugby team may also be the coach and may not have capacity to do baseline assessments, but could still log suspected concussions in the NZR App, thereby engaging players effectively in the process.

## 8 Lesson 4: Building the Right Team to Support Implementation

Embedding concussion guidelines into routine practice in community sport required more than technical expertise or robust evidence; it needed a team structure that could operate effectively withing complexity across sporting, healthcare, and organisational systems. The development and refinement of the CMP was supported by an international, multidisciplinary research team comprising clinicians (1 sports medicine doctor, 5 physiotherapists, 1 athletic therapist), epidemiologists (n = 2), sport coaching researcher (n = 1) and rugby stakeholders (n = 4) from New Zealand (n = 5), Australia (n = 2), South Africa (n = 2), the United States (n = 2), and the United Kingdom (n = 1). The research team worked in close collaboration with community partners from provincial union staff, schools, clubs, GPs, team physiotherapists, coaches, and players. This diversity of perspectives proved critical to identify assumptions, anticipate unintended consequences, and ensure that the pathway remained responsive to real-world community and primary care contexts [37,38].

Bringing together individuals with diverse professional backgrounds and system-level experiences helped ensure that design decisions were relevant and feasible across different healthcare and sporting environments [37]. This was particularly important when adapting processes originally derived from elite sport to the realities of community rugby, volunteer workforces, and primary care constraints. The international composition of the team also strengthened the transferability, supporting reflection on which challenges were context-specific and which were likely to be common across sports and jurisdictions [38].

Equally important was how the research team and community partners functioned. Shared ownership of the project, open communication, and a commitment to iterative learning fostered an environment in which challenges could be surfaced early and adaptations made collaboratively [37]. This approach aligned with principles of systems thinking and implementation science, recognising that interventions evolve through ongoing interaction between people, processes, and context rather than through linear implementation [38].

Our experience reinforces that effective concussion interventions in community sport depend not only on what is implemented, but by who is involved and how they work together. Building and sustaining the “right team”, one that reflects the complexity of the system it seeks to influence, was a foundational element of the CMP's development and offers an important lesson for others undertaking applied injury prevention or surveillance initiatives [38].

## 9 Conclusion

Managing concussions in community sport requires more than disseminating best-practice guidelines; it demands sustained engagement with the environments, cultures, and systems in which those guidelines are enacted. The NZR experience demonstrates that effective concussion management pathways must be treated as living interventions—continuously shaped by stakeholder feedback, resource constraints, healthcare access, and real-world use. Four critical lessons on implementation of concussion guidelines in community sport emerged: 1. Assessment tools must be systematically adapted for community use; 2. Education must evolve alongside the communities it serves; 3. Clear role definition across the pathway is foundational to implementation; 4. Multidisciplinary collaboration and shared ownership are central to sustained effectiveness. These lessons reinforce the importance of moving beyond “one-size-fits-all” solutions and offer practical insights for researchers, governing bodies, and practitioners seeking to translate evidence-informed concussion interventions into sustainable practice across diverse community sport settings.

## Ethics approval and consent to participate

Approval to conduct the research was obtained from the University of Otago Human Ethics Committee (approval 18/087).

## Consent for publication

Not applicable.

## Availability of data and materials

The datasets used and/or analysed during the current study are available from the corresponding author on reasonable request.

## Funding

This work formed part of NZR's National Concussion Strategy and was funded by World Rugby and the Accident Compensation Corporation (ACC) through the RugbySmart programme. The funders did not direct data collection, analysis, interpretation, manuscript preparation, or the decision to submit for publication.

## Declaration of competing interest

The authors declare the following financial interests/personal relationships which may be considered as potential competing interests:

Dr Danielle Salmon reports financial support was provided by World Rugby Limited. Dr Danielle Salmon reports financial support was provided by New Zealand Rugby Foundation. Dr Danielle Salmon reports financial support was provided by Accident Compensation Corp. Dr Danielle Salmon reports a relationship with New Zealand Rugby that includes: employment. Janelle Romanchuk, Marelise Badenhorst, Anja Zoellner, Ian Murphy and Karen Rasmussen were employed or contracted by New Zealand Rugby to support this research. James Brown has also received funding from World Rugby. All other authors, they declare that they have no known competing financial interests or personal relationships that could have appeared to influence the work reported in this paper.

At the time of data collection, DS, JR, MB, IM, KR and AZ were employed by New Zealand Rugby (NZR). Their roles included development and delivery of the CMP. Interpretation and reporting were reviewed by the full multidisciplinary author group, which included authors independent of NZR. All authors reviewed and approved the manuscript.

## Footnotes

Appendix A Supplementary data to this article can be found online at https://doi.org/10.1016/j.jsampl.2026.100156.
