# Supplementary material for: Mahi tahi (working together) and the lessons learned from implementating a concussion management pathway for community rugby in New Zealand: Haerenga (our journey)
Source: JSAMS Plus. 2026 Sep 24;8:100156. doi: 10.1016/j.jsampl.2026.100156 (PMC13635942; doi:10.1016/j.jsampl.2026.100156)
Supplement: Supplementary file 1 [file mmc1.docx]

Appendix 1

**Figure 4:** Concussion cards that were provide to coaches in RugbySmart sessions in tear-off bundles that could be stored in a kit bag and ripped off and provided to a player who had been removed with a suspected concussion

Appendix 2:

**Figure 5:** New Zealand Rugby’s Concussion Fact Sheet which provides graduated return to work/school and sport information.
